# Delegates to the Convention for Revising the National Pharmacopœia

**Published:** 1850-04

**Authors:** Geo. B. Wood

**Affiliations:** Vice President of the Pharm. Convention of 1840; Philada.


DELEGATES TO THE CONVENTION FOR REVISING THE NATIONAL PHARMACOPŒIA.

The following additional names have been reported since the announcement in the last number of the Medical Examiner.

From "the Rhode Island Medical Society," Dr. Joseph Mauran.

From "the Wisconsin State Medical Faculty," George D. Wilbur, M. D.

From "the Medical Society of Delaware," Drs. J. N. Jump, J. D. Perkins, and J. W. Thomson.

Geo. B. Wood,
Vice President of the Pharm. Convention of 1840.

*Philada.*, March 22d, 1850.

